# Supplementary material for: Chromothripsis during telomere crisis is independent of NHEJ, and consistent with a replicative origin
Source: Genome Res. 2019 May;29(5):737–49. doi: 10.1101/gr.240705.118 (PMC6499312; doi:10.1101/gr.240705.118)
Supplement: Supplemental Material [file supp_gr.240705.118_Supplemental_file_1.zip › contigs/annotated_contigs/DB50/contig.2.DB50_length_270_mean_cov_3.35555555556.docx]

**DB50_length_270_mean_cov_3.35555555556**

```
ACTTATATATTTTAATGTTTTGTTTTTTGGG|ACCCAGCCAGATCTGCTGTTAGAGGAGAATTCTTCTGTCCTAGCTGTGTCTCTGTGA
                                >chr4:124573935-124574122 + E=2e-98
TCTGTGGATGAGGGTAGGAAAGCACCACTCAGTCAATTGGGGTGTACAAAATGGAGTTTGAGAAGGTAAGTGATGTTTTCTCCATGCTT

TCTGTTCAAAATTAAGTGCAATTAAATGCTTTTTTTTTTTT|GTTTTGGT|GTTTCACCCTGTTTCCCAGGCTGGAGTG|TAATGTGTA
                                                  >chr2:28028784-28028812 + E=2e-02
ATGTTGG
```
